# Evaluation of the reasons for preferring root canal treatment in mature permanent teeth potentially indicated for pulp preservation: a clinical case/photo-based questionnaire study

**DOI:** 10.1186/s12903-023-03750-0

**Published:** 2023-12-14

**Authors:** Jin-Kyu Yi, An Na Kim, Kyung Hee Kwon

**Affiliations:** 1 Department of Conservative Dentistry, School of Dentistry, Kyung Hee University, Seoul, Korea; 2 Department of Conservative Dentistry, Kyung Hee University Dental Hospital at Gang-dong, Seoul, Korea

**Keywords:** Dental pulp exposure, Pulp preservation procedures, Questionnaire design, Treatment modality for exposed pulp

## Abstract

**Background:**

With advances in pulp preservation procedures (PPP), indications for PPP extend to exposed pulp with symptoms in teeth with carious lesions. Scenario/text-based questionnaire studies report a high preference for PPP for exposed pulp with no pulpal symptoms. However, negative perceptions towards PPP for exposed pulp in carious teeth are prevalent among dentists. Identifying the differences in PPP preference rates in questionnaire studies and actual clinical situations is necessary to determine the current status of PPP. In this study, a clinical case/photo-based design was devised to overcome the limitations of scenario/text-based questionnaires. This study aimed to evaluate the reasons dentists prefer root canal treatment (RCT) in cases where PPP is potentially indicated.

**Methods:**

A questionnaire containing three cases of PPP with successful results was administered to dentists. The cases were selected to elicit comprehensive responses from the dentists. Clinical photos of the pulp exposure sites were presented to dentists without describing the tooth conditions, including the extent of pulp exposure and tooth decay, pulpal surface conditions, or restorability. The questions were focused on the reasons for selecting RCT in cases where was practiced. Questionnaire data were collected using Google e-forms. Chi-squared and Fisher’s exact test (*P* < 0.05) were used for statistical analyses.

**Results:**

Pulpal diagnosis was not a dominant factor in treatment decision-making for pulp exposure during caries removal. Reasons for selecting RCT where PPP was potentially indicated included the event of pulp exposure itself and the dentists’ desire to prevent post-PPP symptoms. Apart from symptomatic pulp, the tooth conditions influenced the establishment of pulpal diagnosis and selection of treatment modality. Moreover, the tooth condition and dentists’ desire for good patient prognosis influenced the negative perceptions towards PPP.

**Conclusions:**

Unfavourable tooth conditions, in association with a desire for preventing post-PPP symptoms, prevent dentists from attempting PPP for pulp exposed during caries removal with no/slight symptoms. Improving negative perceptions towards PPP through accumulation of data on the high success rates of PPP is a prerequisite for achieving widespread application of PPP.

**Supplementary Information:**

The online version contains supplementary material available at 10.1186/s12903-023-03750-0.

## Background

Management of deep carious lesions and pulp exposed during caries removal is a major concern among dentists. In partial caries removal (“selective” or “stepwise”), some carious dentin is left behind to prevent pulp exposure [1, 2]. Total caries removal (“non-selective” or “complete”) is associated with a risk of pulp exposure [3]. Therefore, preservation or elimination of the pulp in carious lesions is a controversial issue. Root canal treatment (RCT) is indicated for symptomatic carious teeth with pulp exposure [4], while vital pulp therapy (VPT) is indicated for mechanically exposed pulp [5]. With advancements in VPT for exposed pulp, pulp-preservation procedures (PPPs) have been introduced to manage pulp exposure in carious teeth with or without symptoms of reversible pulpitis [6–8]. Furthermore, PPPs have been applied to teeth with symptoms indicative of irreversible pulpitis [9, 10]. The term “PPP” has been introduced in this study to emphasize the enhancements in VPT performance for exposed pulp. PPPs include all procedures performed to treat exposed pulp, including visual inspection, resection, haemostasis, and capping. Improved magnifications through the use of dental microscopes or loupes has enhanced the accuracy of the procedures and of visual inspection. Clinical trials on PPP for teeth with irreversible pulpitis indicate its potential benefits over RCT [10, 11]. However, there is no consensus regarding the management of pulp exposed during caries removal, especially in teeth with slight or absent symptoms [12]. Many studies have reported on RCT of teeth with pulp exposure, with and without symptoms of reversible pulpitis [13–15]. On the other hand, studies on PPP have questioned the rationale for removing the exposed pulp in carious teeth with slight or absent symptoms [3, 16].

Questionnaire studies have attempted to elucidate the factors associated with patients and dentists that influence decision-making in cases of deep carious lesions with pulp exposure. Symptoms originating from teeth with vital pulp are considered dominant factors influencing the selection of treatment modality. The development of pulpal symptoms results in invasive treatments, such as RCT [17]. RCT is selected based on the size of pulp exposure [18]. Furthermore, the preference for RCT increases with patient age [14]. The experience and familiarity of dentists with treatment procedures also affect decision-making [19]. The perceived preference for PPP among dentists in clinical settings may be lower than that reported in questionnaire studies. Few studies have investigated why dentists prefer RCT of teeth indicated for PPP. Understanding the factors influencing the low preference for PPP could guide strategies aimed at increasing PPP selection.

Dentists aim to achieve an absence of symptoms following treatment for deep carious lesions [20]. PPP following pulp exposure during caries removal may not be conducive to a favourable prognosis [21]. In this study, it was assumed that factors associated with the teeth such as the size of the pulp exposure site, the condition of the exposed pulp, and the severity of tooth decay, can subjectively influence decision-making [3, 18]. Dentists normally collect information on these factors from clinical photos of exposed pulp in an intuitive manner. Variables based on imaginary scenarios, including presence or absence of symptoms/pulpal exposure, small- or large-sized pulpal exposure sites, and reversible or irreversible pulpitis, provide dichotomic information [14, 15, 18, 20]. Therefore, these types of questionnaires may be ineffective in the identification of factors that affect the decision-making process based on intuition.

To the best of our knowledge, no previous questionnaire-based study has employed clinical images of pulp exposure sites without scenario-based information. We hypothesized that pulp exposure during caries removal and unfavourable tooth conditions such as severe teeth decay, large pulpal exposure sites, and old age, may influence dentists’ decision to exclude PPP as potential treatment. We further hypothesized that these factors also influence the low preference for PPP in cases of pulp exposure with symptoms and even in those without symptoms. This study aimed to determine the reasons dentists prefer RCT in cases potentially indicated for PPP. The participating dentists were presented with three clinical cases of teeth, that had been treated with direct pulp capping (DPC)/pulpotomy, with clinical information, including chief complaints, patient age, clinical photos of pulp exposure sites, pain history, and clinical examinations. Imaginary scenario-based information was excluded from the study to investigate the factors associated with the teeth that exist in latent form and exert their effect in an intuitive manner.

## Methods

All procedures involving human participants were in accordance with the ethical standards of the Institutional Review Board and with the Declaration of Helsinki. This study was approved by the Institutional Review Board (IRB) of Gangdong Kyung-Hee University Hospital (IRB Approval No. KHNMC 2021-03-053). The Review Board waived the requirement for informed consent from the questionnaire participants.

### Case selection for the questionnaire

**Fig. 1:**
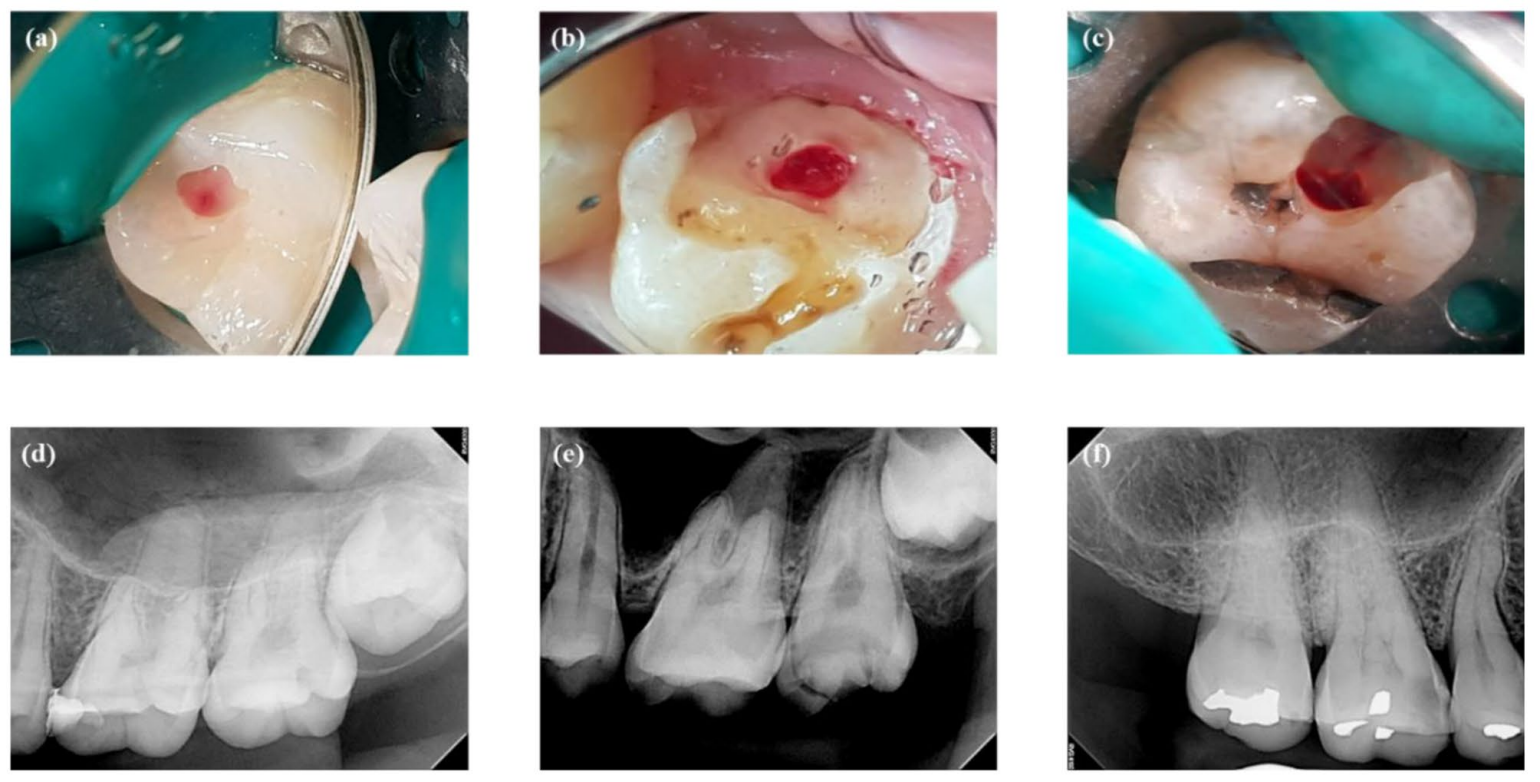
Clinical photos of pulp exposure sites and periapical radiographs. **(a)** Pulp exposure after removal of the secondary caries beneath the old resin restoration in case (1) **(b)** Pulp exposure after caries removal in case (2) The loss of tooth material is severe, and crown restoration is required. **(c)** Pulp exposure after caries removal in case (3) The size of the pulp exposure site and extent of tooth decay are larger than those in case 1. **(d, e, f)** No radiographic changes can be observed in the periapical regions

**Fig. 2:**
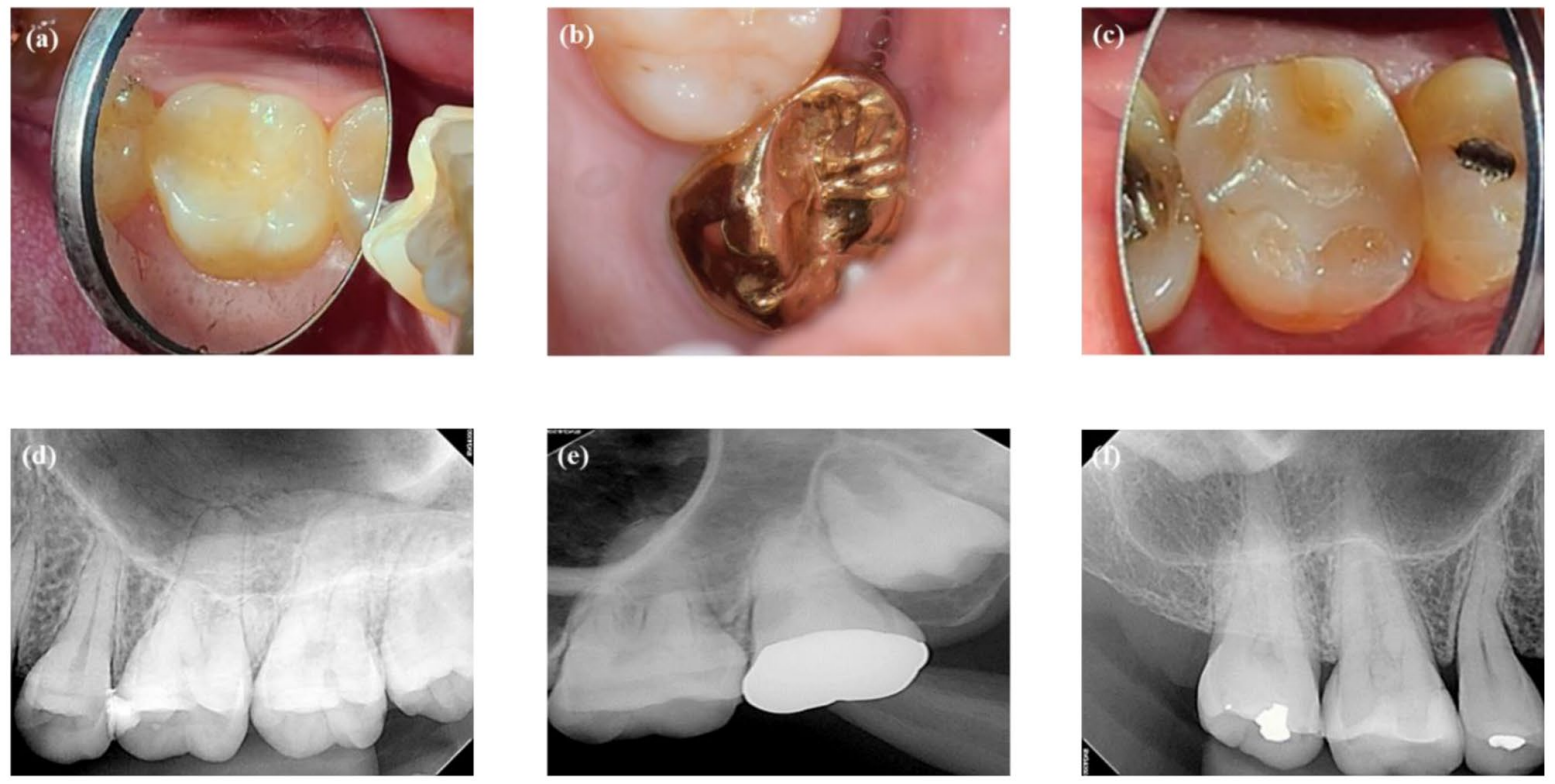
Follow-up evaluation after PPP. **(a)** The tooth in case 1 is restored with composite resin, 32 months after DPC. **(b)** The tooth in case 2 is restored with a gold crown, 37 months after pulpotomy. **(c)** The tooth in case 3 is restored with a resin inlay, 47 months after DPC. **(d, e, f)** No radiographic changes can be observed in the periapical region in cases 1, 2, and 3. Abbreviations: PPP, pulp preservation procedure; DPC, direct pulp capping

Screening for the cases to be included in the questionnaire was performed from the clinical pool of VPT-treated cases in which pulps had been exposed during caries removal and managed by PPP. Two DPC and one pulpotomy cases with successful maintenance after PPP were selected for this study. The screening criteria were as follows: positive response to electric pulp testing (EPT) before DPC/pulpotomy; asymptomatic teeth after DPC/pulpotomy; absence of periapical radiographic changes after DPC/pulpotomy; and difference in one or more screening factors when two cases were compared, including patient age (old versus young), extent of pulp exposure (large versus small), extent of tooth decay (severe versus relatively less severe), and symptoms (indicative of reversible pulpitis versus irreversible pulpitis). To address the limitations, the lack of definitions for such variables, clinical cases with variables showing comparative relations were screened and selected. For instance, in comparing the size of pulpal exposure sites between case 1 and 2, case 2 had a large exposure, while case 1 had a small one (Fig. 1). In three of the cases, the teeth had pulpal exposures with different sizes and different amounts of teeth decay. No periapical radiographic changes were observed in any of the three cases at first examination. Patient in case 2 had spontaneous pain. Slight or absent symptoms were observed in the remaining cases. Cases 1 and 2 were teenagers, whereas case 3 was a 50-year-old. Clinical information of the three cases that were presented to the participants is shown in Table 1. The following results were expected from the questionnaire including the three cases: Many dentists would select RCT for the cases presented in the questionnaire, especially for cases 2 and 3, and these results would be in open contrast to the fact that all three cases had already been treated by PPP. Moreover, all three cases had been followed up for long periods (32, 37, and 47 months for cases 1, 2, and 3, respectively; Fig. 2). It was assumed that precise PPP is associated with VPT success. Therefore, treatment types, DPC or pulpotomy were not considered for case selection in this study.

**Table 1:** Information regarding clinical cases from the questionnaires

Clinical information	Case 1	Case 2	Case 3
Chief complaint	“I have sensitivity and mild pain to cold water”	“I have intermittent spontaneous pain”	“I have a broken molar” (No pain & no sensitivity history)
Age/sex	15/F	16/F	50/M
Tooth number*	14	15	3
Clinical examination	Cold test (+) Percussion (-) Previous resin restoration Secondary caries under restoration	Cold test (-) Percussion (+) Tooth decay on buccal and occlusal surface	Cold test (+) Percussion (-) Dislodgement of restoration Tooth decay on mesial side

* Universal numbering system

### Pulp preservation procedures

Pulp was exposed during caries removal using the complete method. Pulp vitality was preserved by PPP. DPC or pulpotomy were considered after meticulous inspection of the exposed pulpal surface. Pulpal surface had to demonstrate absence of inflammation, avascularity, necrosis, and dentin chips [3]. If necessary, pulp tissue was resected to expose healthy pulpal surface. DPC was performed in cases 1 and 3 during routine caries treatment at the Department of Conservative Dentistry, Kyung Hee University Dental Hospital in Gangdong, Seoul, Korea. Pulpotomy was performed in case 2. At the first visit, caries was removed using the non-selective method. A caries indicator, Seek™ (Ultradent, South Jordan, UT, USA), was used for complete caries removal. Procedures were performed under magnifying loupes (EyeMag®, Zeiss, Mainz, Germany). Bleeding from the exposed pulp was controlled with chlorhexidine-soaked cotton pellets. The exposed pulpal surface was examined, and the absence of inflamed tissue or dentine chips was confirmed. Biodentine (Septodont, Saint-Maur-des-Fossés Cedex, France) was used as capping material. The exposed pulp and tooth cavity were managed using the “total capping” method. Briefly, Biodentine (Septodont) was prepared according to the manufacturer’s protocols and applied to the exposed pulpal surface. Care was taken to generate gentle and hermetic contact with the exposed pulp. Total capping was performed after pulpal application to fill the entire cavity to the level of the occlusal surface. Excessive material was removed to adjust the occlusion after setting of the capping material. At the second visit, the patient’s symptoms were examined. The prerequisites for the final restoration were absence of symptoms and positive response to the EPT. Teeth were restored with composite resin in case 1, gold crown in case 2, and resin inlay in case 3. The total capping method allowed a Biodentine (Septodont) base for the final restoration. The two-step procedure was performed to increase the mechanical strength of the capping material [22, 23].

### Questionnaire procedures

The questionnaire contained items on patients’ clinical information, including chief complaints, age, sex, and clinical examinations (Table 1). Clinical photos of pulp exposure sites and periapical radiographs were also provided to the dentists (Fig. 1). Any information that could discourage subjective reasoning processes by the dentists, including pulpal diagnosis, judgements of pulpal surface conditions or degree of teeth decay, and restorability of the teeth, was excluded. The questionnaire included questions related to pulpal diagnosis, treatment modalities, restoration type, reasons for selecting RCT, and reasons for the differences in treatments between cases (Tables 2 and 3). The dentist-associated factors were years of clinical experience and postgraduate education. Questionnaire form on first survey was listed in additional file (Additional file 1).

**Table 2:** Summary of questions and responses relating to the data presented in this article

Question topics	Variables	Case 1 *n* (%)	Case 2 *n* (%)	Case 3 *n* (%)
Diagnosis	Reversible pulpitis	40/55 (72.73)	3/55 (5.45)	20/55 (36.36)
	Irreversible pulpitis	14/55 (25.45)	44/55 (80.00)	31/55 (56.36)
	Pulp necrosis	0/55 (0.00)	3/55 (5.45)	2/55 (3.64)
	Chronic apical periodontitis	0/55 (0.00)	4/55 (7.27)	0/55 (0.00)
	Others	1/55 (1.82)	1/55 (1.82)	2/55 (3.64)
Treatment modality	DPC	53/98 (54.08)	2/98 (2.04)	6/98 (6.12)
	Partial pulpotomy	4/98 (4.08)	2/98 (2.04)	0/98 (0)
	Full pulpotomy	3/98 (3.06)	4 /98 (4.08)	2/98 (2.04)
	RCT	38/98 (38.78)	90/98 (91.84)	90/98 (91.84)
Restoration	Direct filling	46/98 (46.94)	1/98 (1.02)	1/98 (1.02)
	Inlay or onlay	14/98 (14.28)	1/98 (1.02)	5/98 (5.10)
	Crown	38/98 (38.78)	96/98 (97.96)	92/98 (93.88)
Reasons of selecting RCT	Pulp is exposed after caries removal	15/38 (39.47)	19/90 (21.11)	25/90 (27.78)
	Large pulp exposure site	0/38 (0.00)	17/90 (18.89)	11/90 (12.22)
	Pulpal diagnosis is irreversible pulpitis	2/38 (5.26)	26/90 (28.89)	9/90 (10.00)
	Unfamiliarity with the procedures of DPC or pulpotomy	0/38 (0.00)	0/90 (0.00)	0/90 (0.00)
	Prevention of symptoms after performing DPC/pulpotomy	14/38 (36.84)	12/90 (13.33)	12/90 (13.33)
	Prevention of symptoms after practicing crown restoration	6/38 (15.79)	16/90 (17.78)	30/90 (33.33)
	Others	1/38 (2.63)	0/90 (0)	3/90 (3.33)

RCT, root canal treatment; DPC, direct pulp capping

**Table 3:** Reasons for selecting RCT: recategorized variables

Recategorized variables	Case 1 *n* (%)	Case 2 *n* (%)	Case 3 *n* (%)
Event of pulp exposure itself (Pulp is exposed after caries removal and large pulp exposure site)	15/38 (39.47)	36/90 (40.00)	36/90 (40.00)
Pulpal diagnosis is irreversible pulpitis	2/38 (5.26)	26/90 (28.89)	9/90 (10.00)
Unfamiliarity with the procedures of DPC or pulpotomy	0/38 (0.00)	0/90 (0.00)	0/90 (0.00)
Pursuit of symptom-free after treatment (Prevention of symptoms after performing DPC/pulpotomy and crown restoration)	20/38 (52.63)	28/90 (31.11)	42/90 (46.67)
Others	1/38 (2.63)	0/90 (0.00)	3/90 (3.33)

RCT, root canal treatment; DPC, direct pulp capping

This study was conducted in cooperation with two local dental associations, Hanam City Dental Association and Gangdong-Gu Dental Association. Both associations were selected owing to their proximity and an endodontic referral relationship with Kyung Hee University Dental Hospital, Gangdong. Because local Korean dental associations do not differentiate between different types of dentists, the questionnaire results were thought to represent those of the average Korean dentist. Questionnaires were distributed as Google e-forms to the members via email by the executive branches of the two dental associations. No personal information, including name, phone number, or email address, was collected or provided. Fifty-five dentists responded to the first survey and the response rate was 19.4%. The identified reasons for the low response rate on the first survey were difficulties in answering the questions. Therefore, questionnaire form was modified to secure numbers of participants by increasing respondents’ easy in answering to the questions. Questions regarding pulpal diagnosis were excluded in the questionnaire. Information that all the three cases had been treated by VPT was provided on second survey. The second survey was performed using a modified questionnaire to increase study participation. Questionnaire form on second survey was listed in additional file (Additional file 2).

### Statistical analyses

Data with categorical variables were analysed using the chi-squared and Fisher’s exact tests (P < 0.05). RStudio (version 4.2.2, RStudio, Inc., Boston, MA, USA) was used for the statistical analysis.

## Results

The questionnaire was sent to 283 dentists, and the response rate was 34.9%. The number of responses was 55 and 44 for the first and second requests, respectively. One response was deemed not appropriate for statistical analyses, and therefore 98 responses were included in the analysis. The response results are presented in Table 2. Pulpal diagnosis was evaluated only in the first questionnaire. In case 1, the rate of diagnosis of reversible pulpitis (72.73%) was higher than that of irreversible pulpitis (25.45%). In case 2, most dentists (80.00%) determined that the pulpal conditions were irreversible. The rate of an irreversible pulpitis diagnosis was more than two times higher in case 3 (56.36%) compared to case 1 (25.45%). Ninety dentists (91.84%) selected RCT as treatment modality in cases 2 and 3. DPC was selected significantly less frequently in cases 2 and 3 than in case 1. Dentists preferred crown restoration in cases 2 and 3. Variables in the reasons for selecting RCT in Table 2 were rearranged (Table 3). “Pulp exposure after caries removal” and “large pulp exposure site” were included in the same category, “event of pulp exposure itself”, whereas “prevention of symptoms after performing DPC/pulpotomy” and “prevention of symptoms after practicing crown restoration” were included in the “pursuit of symptom-free teeth after treatment” category.

The reasons for selecting RCT were analysed according to the recategorized variables. The most common reasons for selecting RCT were the “pursuit of symptom-free teeth after treatment” in cases 1 (52.63%) and 3 (46.67%), and “event of pulp exposure itself” in case 2 (40.00%). The second most common reasons were “event of pulp exposure itself” in cases 1 (39.47%) and 3 (40.00%) and the “pursuit of symptom-free teeth after treatment” in case 2 (31.11%). Reasons for altering the treatment modality are presented in Table 4. The most common reason for proposing different treatment modalities in cases 1 and 2 was a difference in the size of the pulp exposure site (53.13%), followed by a different pulpal diagnosis (34.38%). Patient age (36.07%) and size of the pulp exposure site (36.07%) were the main reasons for selecting different treatments in cases 1 and 3. Further, the size of the pulp exposure site (41.38%) was the most common reason for differences in the proposed treatments in cases 2 and 3, followed by patient age (31.03%).

**Table 4:** Reasons for altering treatment modality

Variables	Questions		
	if the treatment modality was different between case 1 and case 2, what is the reason? *n* (%)	if the treatment modality was different between case 1 and case 3, what is the reason? *n* (%)	if the treatment modality was different between case 2 and case 3, what is the reason? *n* (%)
Patient’s age	5/64 (7.81)	22/61 (36.07)	9/29 (31.03)
Different pulpal diagnosis/condition	22/64 (34.38)	7/61 (11.48)	5/29 (17.24)
Different size of the pulp exposure site	34/64 (53.13)	22/61 (36.07)	12/29 (41.38)
Different restoration type	3/64 (4.69)	6/61 (9.84)	1/29 (3.45)
Others	0/64 (0.00)	4/61 (6.56)	2/29 (6.90)

In case 3, the pattern of selection of treatment modality depended on the years of clinical experience (Table 5). Selection patterns of treatment modalities differed between cases 1 and 3, and between cases 2 and 3 (P < 0.05). The reasons for selecting RCT differed between cases 1 and 3, and between cases 2 and 3 (P < 0.05). Selection patterns of treatment modality, final restoration type, and reason for selecting RCT showed no difference based on the dentists’ postgraduate education (P > 0.05).

**Table 5:** Difference in the selection of treatment modalities according to years of clinical experience

Clinical cases	Treatment modality	Total *N*	0–9 years Total *N*: 51 *n* (%)	10–19 years Total *N*: 75 *n* (%)	20–29 years Total *N*: 81 *n* (%)	Over 30 years Total *N*: 87 *n* (%)	p-value*
**Case 1**	DPC	53	10 (58.82)	9 (36.00)	18 (66.67)	16 (55.17)	0.2191
	Pulpotomy	7	2 (11.76)	1 (4.00)	1 (3.70)	3 (10.34)	
	Pulpectomy	38	5 (29.41)	15 (60.00)	8 (29.63)	10 (34.48)	
**Case 2**	DPC	2	0 (0.00)	0 (0.00)	0 (0.00)	2 (6.90)	0.4808
	Pulpotomy	6	0 (0.00)	1 (4.00)	2 (7.41)	3 (10.34)	
	Pulpectomy	90	17 (100.00)	24 (96.00)	25 (92.59)	24 (82.76)	
**Case 3**	DPC	6	1 (5.88)	0 (0.00)	0 (0.00)	5 (17.24)	0.0345
	Pulpotomy	2	0 (0.00)	0 (0.00)	1 (3.70)	1 (3.45)	
	Pulpectomy	90	16 (94.12)	25 (100.00)	26 (96.30)	23 (79.31)	

*Fisher’s exact test, DPC, direct pulp capping

## Discussion

Questionnaire studies have demonstrated that PPP is preferred over RCT for the management of pulp exposure after caries removal without symptoms [13, 16]. However, the perceived preference for PPP in clinical settings may be lower than that reported by questionnaire studies. Therefore, experimental questionnaires should be revised to more effectively uncover the reasons for this discrepancy. Questionnaire studies provide information about the clinical considerations associated with decision-making, including pulpal symptoms, patient age, and size of the pulp exposure site [14, 24, 25]. Scenarios involving symptomatic or asymptomatic teeth, reversible or irreversible pulpitis, and young or old patients, can guide dichotomic or univariate decisions instead of relying on a comprehensive subjective process. Tooth conditions, including pulp exposure, the size of the pulp exposure site, and the extent of tooth decay, influence the selection of the treatment modality by the dentist in a subjective manner.

Describing or quantifying the factors associated with tooth condition in text form is difficult. Clinical photos of pulp exposure sites provide information regarding the tooth condition in image form with latent information, including the size of the exposed pulpal site, colour of the blood, extent of tooth decay, and surface condition of the exposed pulp (Fig. 1). In this study, descriptions of the latent information in the clinical photos were excluded to allow a subjective reasoning process regarding the factors associated with tooth condition during treatment, thereby avoiding univariate decision-making. Clinical case/photo-based questionnaires are effective in terms of reflecting actual clinical situations.

Symptoms originating from teeth with vital pulp affect the establishment of the pulpal diagnosis [17]. In case 2, the presence of intermittent spontaneous pain may have elicited the high rate of diagnosis of irreversible pulpitis (80.00%; Table 2). Although there were insufficient data indicative of irreversible pulpitis in cases 1 and 3, the diagnosis rates of irreversible pulpitis were 25.45% and 56.36%, respectively. A diagnosis of irreversible pulpitis could be made without pulpal symptoms, as shown in cases 1 and 3. The symptoms in case 3 were similar to those in case 1, but the rate of diagnosis of irreversible pulpitis was higher in case 3 than in case 1. When tooth conditions in cases 1 and 3 were compared, the tooth in case 3 had unfavourable conditions, including older patient age, severe tooth decay, and large pulp exposure site (Fig. 1; Table 1). The higher rate of diagnosis of irreversible pulpitis in case 3 than that in case 1 could be explained by the complicated conditions of the tooth in case 3. These data imply that symptoms originating from teeth with vital pulp were not dominant factors in pulpal diagnosis.

The rate of RCT selection as treatment modality in cases 1 (38.78%) and 3 (91.84%) demonstrated different perspectives regarding treatment decision-making under unfavourable tooth conditions. Pulpal diagnosis was not a dominant factor for selecting RCT, as observed for case 3. Obtaining different rates of irreversible pulpitis diagnosis and PPP selection between cases 1 and 3 would not be possible under scenario/text-based designs. Scenario/text-based information would be ineffective in conveying the different factors associated with tooth conditions in cases 1 and 3, and thereby affect dentists’ preference for selecting PPP in a subjective manner.

The rate of irreversible pulpitis diagnosis was lower in case 3 (56.36%) than in case 2 (80.00%), but the rate of RCT selection was similar in both cases (91.84%). Patient age, presence of spontaneous pain, and extent of tooth decay differed between cases 2 and 3 (Fig. 1; Table 1). Although the tooth in case 3 did not show spontaneous pain and its condition was more favourable compared to case 2, the preference for RCT was the same as that in case 2. Pulpal diagnosis did not explain the preference for RCT in case 3. These results imply that the perceptions to the tooth condition in association with the desire to prevent post-PPP symptoms influence the preference for RCT. The preference for PPP in carious teeth with an exposed pulp and slight or absent symptoms, as observed in case 3, would be low in actual clinical situations.

In this study, the participating dentists preferred RCT of teeth with an exposed pulp and mild symptoms to achieve symptom-free teeth after treatment (Table 3). This study also revealed the dentists’ negative perceptions towards PPP for managing teeth with an exposed pulp with reversible pulpitis. The difference in the preferred treatment modality according to the years of clinical experience for case 3 (Table 5) may indicate an acquired perception toward treating exposed pulp in carious lesions. Experienced dentists may prefer RCT over PPP for teeth with an exposed pulp and reversible pulpitis to prevent post-PPP symptoms. The need for an invasive restoration, due to severe tooth decay, can result in the selection of RCT owing to the dentists’ desire for good clinical results. Analysis of the reasons for RCT preference and alteration of treatment modality demonstrated the negative perceptions of dentists towards pulp exposure during caries removal (Tables 2 and 3, and 4). Negative perceptions to the exposed pulp in carious lesions were associated with a preference for RCT and avoidance of PPP.

Collectively, this study suggests the following: First, clinical case/photo-based questionnaires can effectively demonstrate a preference for PPP in cases of exposed pulp with slight or absent symptoms in real-world clinical settings. Second, pulpal diagnosis and selection of the treatment modality are multifactorial decision-making processes. Third, symptoms originating from teeth with vital pulp might not be a dominant factor in the decision-making process. Fourth, dentists’ perceptions of the tooth condition, in association with the desire to prevent post-PPP symptoms, may influence the pulpal diagnosis and preference for RCT. With the development of enhanced PPP protocols, studies on PPP have demonstrated increased success rates and extended indications for the procedure [16, 26]. However, PPP is still recognized as an unpredictable treatment for the management of exposed pulp in carious lesions. This study presents the factors associated with the selection of RCT for teeth with exposed pulp with mild or absent symptoms that could be treated with PPP. We used cases in which PPP was performed to demonstrate its potential clinical utility, regardless of whether dentists preferred RCT. All cases in this study showed good clinical results at the follow-up examination (Fig. 2). These results suggest the need to improve clinicians’ attitudes towards performing PPP for exposed pulp in carious lesions.

This study introduced enhanced questionnaire design containing information without subjective assessments provided by researchers, such as pulpal diagnosis. Clinical case/photo-based questionnaires have advantages in conveying latent information. Clarifying different perceptions to PPPs according to the intuitive recognition of teeth conditions was possible due to selecting clinical cases with contrasting variables. Investigation of dentists’ perceptions toward PPP may imply strategy for widespread application of PPP.

## Conclusions

A clinical case/photo-based design is an effective method for developing experimental questionnaires on PPP. The tooth condition in combination with the desire to prevent post-PPP symptoms influenced the dentists’ preference for RCT of teeth with an indication for PPP. Counteracting negative perceptions towards PPP by accumulating data of its high success rates is a prerequisite for widespread adoption of the procedure in clinical settings.

### Electronic supplementary material

Below is the link to the electronic supplementary material.

**Additional file 1**: Questionnaire form on first survey. A document containing questions and clinical information for a questionnaire that was used on the first survey.

**Additional file 2**: Questionnaire form on second survey. A modified questionnaire form for second survey.

## Data Availability

The datasets used and/or analysed during the current study are available from the corresponding author on reasonable request.

## Abbreviations

DPC: Direct pulp capping
EPT: Electric pulp testing
PPP: Pulp preservation procedures
RCT: Root canal treatment
VPT: Vital pulp therapy
