# Supplementary material for: Evaluation of the reasons for preferring root canal treatment in mature permanent teeth potentially indicated for pulp preservation: a clinical case/photo-based questionnaire study
Source: BMC Oral Health. 2023 Dec 14;23:1003. doi: 10.1186/s12903-023-03750-0 (PMC10722753; doi:10.1186/s12903-023-03750-0)
Supplement: Supplementary file 1 — Additional file 1: Questionnaire form on first survey. A document containing questions and clinical information for a questionnaire that was used on the first survey. [file 12903_2023_3750_MOESM1_ESM.docx]

Thank you for your participating this questionnaire. This questionnaire aims to investigate current status of vital pulp therapy at local dental clinics. Your responses will be utilized exclusively for statistical purposes in academic research. Questionnaire comprised three clinical cases. There are no assigned answers. Please feel free to response to the questions based on your judgement.

**Baseline questions**

1. Age:

2. Year of graduation of dental school:

3. Name of Dental School:

4. Certification of Specialist or Degree of post-graduate educations:

**Case 1**

A 15-year-old girl visited dental hospital with a chief complaint of hypersensitivity or mild pain to cold water in the upper left molar region. Upper left first molar (#26 tooth) was sensitive to cold test. Tooth demonstrated negative response to percussion and absence of mobility. Secondary caries was suspicious beneath previous resin restoration (left fig.). Secondary caries was identified during removal of resin restoration. Pulp was exposed during caries removal (right fig.).


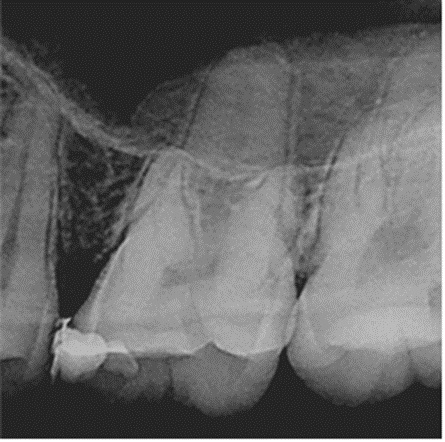

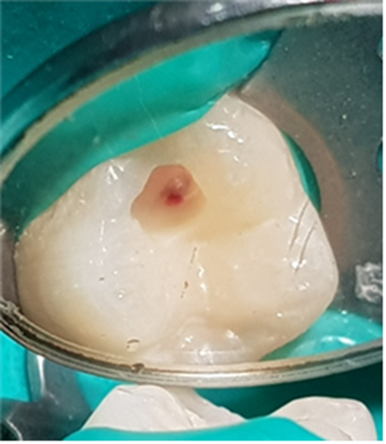


1. What is a pulpal or periapical diagnosis?

□ Reversible pulpitis

□ Irreversible pulpitis

□ Pulp necrosis

□ Chronic apical periodontitis

□ Others (__________________________________)

2. What is your treatment modality for exposed pulp?

① Direct pulp capping

② Partial pulpotomy

③ Full pulpotomy

④ Root canal treatment

3-1. (If you chose one among ①, ②, or ③ for treatment of exposed pulp in question 2)

What is your choice for pulp capping material?

□ Calcium hydroxide

□ MTA

□ Biodentine^TM^

□ Zinc oxide eugenol

□ ETC (__________________________________)

3-2. (If you chose ④ for treatment of exposed pulp in question no. 2)

What’s the reason for selecting root canal treatment?

□ Pulp is exposed after caries removal

□ Large pulp exposure site

□ Pulpal diagnosis is irreversible pulpitis

□ Unfamiliarity with the procedures of DPC or pulpotomy

□ Prevention of symptoms after performing DPC/pulpotomy

□ Prevention of symptoms after practicing crown restoration

□ Others (__________________________________)

4. What is your restoration plan after management of exposed pulp?

□ Direct resin filling

□ Inlay or onlay

□ Crown

**Case 2**

A 16-year-old girl visited dental hospital with a chief complaint of intermittent spontaneous pain in upper left molar region. Caries was identified on buccal and occlusal surface in upper left second molar (#27 tooth). Intraoral radiograph demonstrated deep caries in upper left second molar (left fig.). Tooth was negative to cold test and positive to percussion. Mobility was absent. Pulp was exposed during caries removal (right fig.).


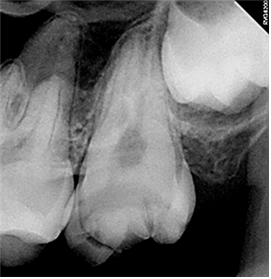

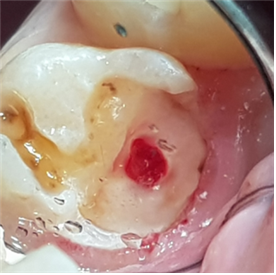


1. What is a pulpal or periapical diagnosis?

□ Reversible pulpitis

□ Irreversible pulpitis

□ Pulp necrosis

□ Chronic apical periodontitis

□ Others (__________________________________)

2. What is your treatment modality for exposed pulp?

① Direct pulp capping

② Partial pulpotomy

③ Full pulpotomy

④ Root canal treatment

3-1. (If you chose one among ①, ②, or ③ for treatment of exposed pulp in question 2)

What is your choice for pulp capping material?

□ Calcium hydroxide

□ MTA

□ Biodentine^TM^

□ Zinc oxide eugenol

□ ETC (__________________________________)

3-2. (If you chose ④ for treatment of exposed pulp in question no. 2)

What’s the reason for selecting root canal treatment?

□ Pulp is exposed after caries removal

□ Large pulp exposure site

□ Pulpal diagnosis is irreversible pulpitis

□ Unfamiliarity with the procedures of DPC or pulpotomy

□ Prevention of symptoms after performing DPC/pulpotomy

□ Prevention of symptoms after practicing crown restoration

□ Others (__________________________________)

4. What is your restoration plan after management of exposed pulp?

□ Direct resin filling

□ Inlay or onlay

□ Crown

5. If the treatment modality was different between case 1 and case 2, what is the reason?

□ Patient’s age

□ Different pulpal diagnosis/condition

□ Different size of the pulp exposure site

□ Different restoration type

□ Others (__________________________________)

**Case 3**

A 50-year-old man visited dental hospital with a chief complaint of “I have a broken molar in upper right region”. Previous amalgam restoration was exfoliated and secondary caries was observed in upper right first molar (#16 tooth). Intraoral radiograph demonstrated deep cavity in upper right first molar (left fig.). Tooth revealed no pain/hypersensitivity history. At the clinical examination, tooth was hypersensitive to cold test and negative to percussion. Mobility was absent. Pulp was exposed during caries removal (right fig.).


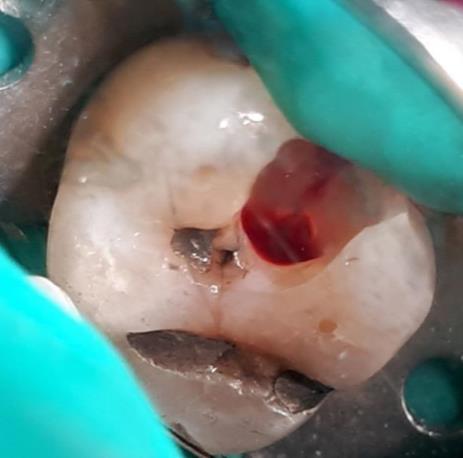

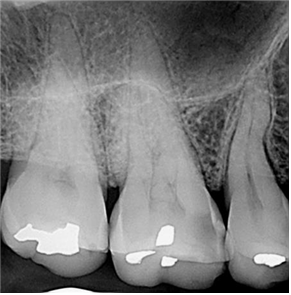


1. What is a pulpal or periapical diagnosis?

□ Reversible pulpitis

□ Irreversible pulpitis

□ Pulp necrosis

□ Chronic apical periodontitis

□ Others (__________________________________)

2. What is your treatment modality for exposed pulp?

① Direct pulp capping

② Partial pulpotomy

③ Full pulpotomy

④ Root canal treatment

3-1. (If you chose one among ①, ②, or ③ for treatment of exposed pulp in question 2)

What is your choice for pulp capping material?

□ Calcium hydroxide

□ MTA

□ Biodentine^TM^

□ Zinc oxide eugenol

□ ETC (__________________________________)

3-2. (If you chose ④ for treatment of exposed pulp in question no. 2)

What’s the reason for selecting root canal treatment?

□ Pulp is exposed after caries removal

□ Large pulp exposure site

□ Pulpal diagnosis is irreversible pulpitis

□ Unfamiliarity with the procedures of DPC or pulpotomy

□ Prevention of symptoms after performing DPC/pulpotomy

□ Prevention of symptoms after practicing crown restoration

□ Others (__________________________________)

4. What is your restoration plan after management of exposed pulp?

□ Direct resin filling

□ Inlay or onlay

□ Crown

5. If the treatment modality was different between case 1 and case 2, what is the reason?

□ Patient’s age

□ Different pulpal diagnosis/condition

□ Different size of the pulp exposure site

□ Different restoration type

□ Others (__________________________________)

Thank you for your participation.
